# Molecular Chaperones: A Double-Edged Sword in Neurodegenerative Diseases

**DOI:** 10.3389/fnagi.2020.581374

**Published:** 2020-10-06

**Authors:** Jessica Tittelmeier, Eliana Nachman, Carmen Nussbaum-Krammer

**Affiliations:** German Cancer Research Center (DKFZ), Center for Molecular Biology of Heidelberg University (ZMBH), DKFZ-ZMBH Alliance, Heidelberg, Germany

**Keywords:** neurodegenarative diseases, prion-like spreading, proteostasis, molecular chaperones and Hsps, disaggregation

## Abstract

Aberrant accumulation of misfolded proteins into amyloid deposits is a hallmark in many age-related neurodegenerative diseases, including Alzheimer’s disease (AD), Parkinson’s disease (PD), Huntington’s disease (HD), and amyotrophic lateral sclerosis (ALS). Pathological inclusions and the associated toxicity appear to spread through the nervous system in a characteristic pattern during the disease. This has been attributed to a prion-like behavior of amyloid-type aggregates, which involves self-replication of the pathological conformation, intercellular transfer, and the subsequent seeding of native forms of the same protein in the neighboring cell. Molecular chaperones play a major role in maintaining cellular proteostasis by assisting the (re)-folding of cellular proteins to ensure their function or by promoting the degradation of terminally misfolded proteins to prevent damage. With increasing age, however, the capacity of this proteostasis network tends to decrease, which enables the manifestation of neurodegenerative diseases. Recently, there has been a plethora of studies investigating how and when chaperones interact with disease-related proteins, which have advanced our understanding of the role of chaperones in protein misfolding diseases. This review article focuses on the steps of prion-like propagation from initial misfolding and self-templated replication to intercellular spreading and discusses the influence that chaperones have on these various steps, highlighting both the positive and adverse consequences chaperone action can have. Understanding how chaperones alleviate and aggravate disease progression is vital for the development of therapeutic strategies to combat these debilitating diseases.

## Introduction

A common feature in many neurodegenerative diseases, including Alzheimer’s disease (AD), Parkinson’s disease (PD), Huntington’s disease (HD), amyotrophic lateral sclerosis (ALS), and prion diseases is the age-related formation of amyloid deposits (Chiti and Dobson, 2017). Each disorder is characterized by the misfolding of one or more specific proteins: amyloid-β (Aβ) and Tau (MAPT) in AD, α-synuclein (α-syn/SNCA) in PD, Huntingtin (HTT) in HD, superoxide dismutase 1 (SOD1), TAR DNA binding protein 43 (TDP-43/TARDBP), FUS RNA-binding protein (FUS) and dipeptide repeat proteins (DPRs) translated from C9orf72-SMCR8 complex subunit (C9orf72) in ALS, and the prion protein (PrP/PRNP) in prion diseases (Dobson, 2017; Eisenberg and Sawaya, 2017). Despite having different structures and functions under physiological conditions, under disease conditions, these proteins adopt a β-sheet-rich conformation with a strong tendency to form highly ordered amyloid fibrils. These fibrils can act as pernicious templates for the native monomeric form of the respective protein to misfold into the amyloid conformation and incorporate into the growing fibrils, which eventually accumulate into large intra- and/or extracellular deposits characteristic for the respective neurodegenerative diseases (Jucker and Walker, 2013).

Protein aggregates usually arise from the failure of the protein quality control (PQC) machinery that maintains cellular protein homeostasis (proteostasis). Molecular chaperones are key components of the PQC network and support cellular proteostasis by regulating the folding of nascent polypeptides, the re-folding of aberrant proteins, or their removal by degradation *via* the ubiquitin-proteasome system (UPS) or autophagy (Bukau et al., 2006; Kampinga and Craig, 2010). When a protein escapes these (re)-folding or clearance mechanisms, misfolded forms accumulate and eventually aggregate (Hartl et al., 2011). An age-related decline in the capacity of the PQC machinery appears to result in a proteostasis collapse (Ben-Zvi et al., 2009), which in turn allows the manifestation of diseases associated with protein misfolding, such as the diseases mentioned above. On a positive note, the age-dependent accumulation of amyloid deposits in neurodegenerative diseases suggests that in younger individuals there are PQC pathways active that can prevent aggregation. Chaperones are key regulators of amyloid formation since they monitor and prevent the misfolding and aggregation of proteins (Kampinga and Bergink, 2016; Wentink et al., 2019). Here, we will highlight the complex ways in which chaperones influence the different stages of prion-like propagation of proteins associated with the most prevalent neurodegenerative diseases. This will contribute to a better understanding, not only of which chaperones could be selected for drug development, but also of when to target these chaperones.

## Prion-Like Propagation of Protein Misfolding in Neurodegenerative Diseases

The propensity of a protein aggregate to act as a template or “nucleus” or “seed” to promote the aggregation of its native form is central to the prion hypothesis (Prusiner et al., 1998). Prions (proteinaceous infectious particles) are the causative agent in prion diseases including bovine spongiform encephalopathy (BSE), chronic wasting disease (CWD), and scrapie (Prusiner et al., 1998). In prion diseases, disease-associated PrP^Sc^ can propagate itself by templating the conversion of the endogenous PrP^C^ from its normal helical into a β-sheet-rich amyloid conformation (Prusiner, 1998; Glynn et al., 2020). These diseases are truly infectious as they can spread within and between species. While misfolded proteins associated with other neurodegenerative diseases do not seem to be naturally transmitted between individuals, they share many properties of prions, such as the ability to self-propagate, spread from cell to cell, and subsequently induce aggregation of the same protein in neighboring cells (Walker and Jucker, 2015). They are often referred to as “prion-like” to differentiate them from truly infectious prions, but to emphasize the strong similarities concerning the propagation process (Jaunmuktane and Brandner, 2019).

**Figure 1:**
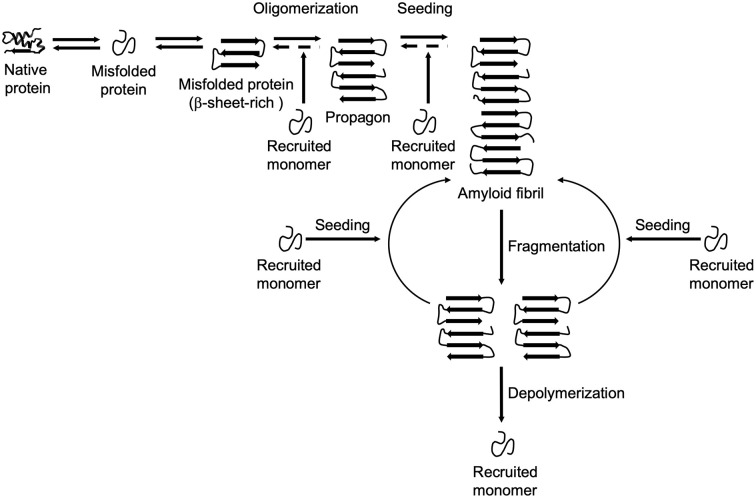
Amyloid formation and propagation at the molecular level. Native proteins oligomerize after adopting an abnormal β-sheet-rich fold, eventually forming a propagon. Propagons are specific units that can recruit and incorporate native monomers, which allows them to grow into amyloid fibrils. Fragmentation events can lead to complete depolymerization into monomers or to the formation of new propagons that in turn provide more ends for recruiting monomers.

**Figure 2:**
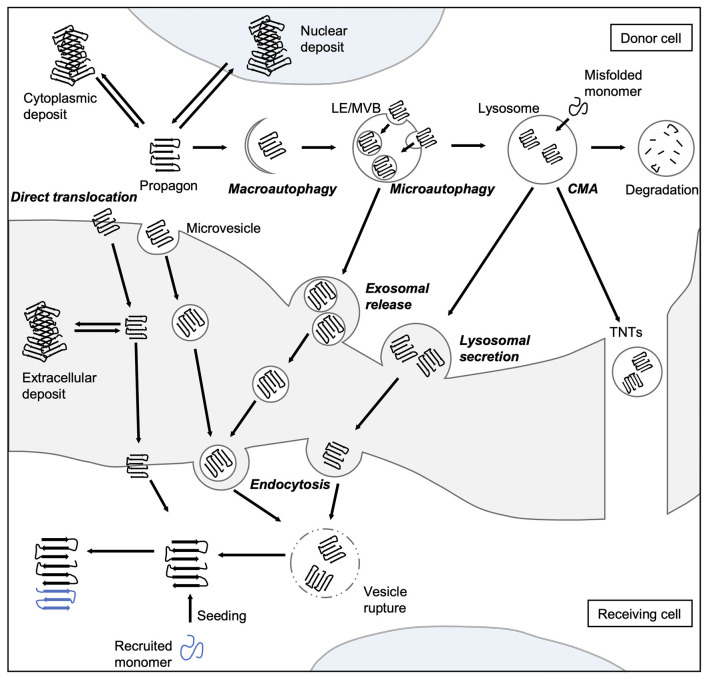
Spreading routes of amyloidogenic proteins. The intercellular transmission of disease proteins can occur *via* several pathways. Some substrates can be translocated directly across the plasma membrane or shed *via* microvesicles. Misfolded proteins can be also targeted by all branches of autophagy, and thereby enter the endo-lysosomal system. Aggregates can either be engulfed *via* bulk or selective (involving adaptor proteins, such as sequestosome 1 (SQSTM1/p62) macroautophagy or taken up into LEs and MVBs through microautophagy. Abnormal proteins may also be directly ingested by lysosomes as a result of CMA. Degradation-resistant aggregates may be released into the extracellular space by lysosomal fusion with the plasma membrane or transported into neighboring cells *via* TNTs. Endocytosis mediates the uptake of either free protein or extracellular vesicles containing the disease-associated protein. To be released into the cytosol of the receiving cell, misfolded proteins can induce endocytic vesicle rupture. Released proteins can then recruit monomers and catalyze their incorporation, which eventually leads to the formation of amyloid aggregates in the receiving cell. CMA, chaperone-mediated autophagy; LE, late endosome; MVB, multivesicular body; TNT, tunneling nanotube.

The formation and propagation of amyloids involve several critical steps. The initial conformational rearrangement to an abnormal β-sheet-rich fold favors the assembly of individual proteins into an oligomer (Figure 1). The generation of a propagon, a unit with a seeding-competent conformation and size that can self-replicate, is considered the rate-limiting event in amyloid formation (Cox et al., 2003; Iljina et al., 2016; Meisl et al., 2017). Elongation of protofibrillar species, or the templated addition of misfolded proteins, proceeds relatively fast. Although an amyloid fibril is energetically very stable, there is still an equilibrium between different oligomeric, protofibrillar, and fibrillar protein species (Carulla et al., 2005; Baldwin et al., 2011). Fibril growth is further accelerated by secondary nucleation events along the fibril surface and by fragmentation (Törnquist et al., 2018). The latter event promotes amyloid growth by producing more fibril ends to which monomers can be added (Knowles et al., 2009). Also, propagons are further able to spread *via* multiple routes outlined in Figure 2 and template the aggregation of like proteins in neighboring cells.

This intra- and intercellular propagation of aggregated material seems to underlie the characteristic progressive spreading of pathology in prion and prion-like diseases (Jucker and Walker, 2013). Since molecular chaperones can protect cells from harmful protein aggregates, at least at a young age, they are gaining increasing attention in current research to develop intervention strategies.

## The Role of Chaperones in Prion-Like Propagation

Molecular chaperones, first identified as heat shock proteins (Hsps), help fold newly synthesized proteins, inhibit and reverse the misfolding and aggregation, and assist in the degradation of terminally misfolded proteins, thereby maintaining cellular proteostasis under physiological and stress conditions (Klaips et al., 2018). Chaperones recognize hydrophobic motifs in misfolded proteins that are usually hidden in their native folded state. A mere binding activity is considered a holdase function that does not require ATP. However the folding and refolding of proteins often rely on an ATP-dependent cycle that allows the repeated binding and release of chaperones, thereby facilitating (re)-folding processes (Mayer and Bukau, 2005; Liberek et al., 2008). The latter activity is performed by ATP-dependent chaperones, which often require specific co-chaperones that are responsible for regulating the ATP cycle (binding, hydrolysis, and release) and in turn influence substrate specificity and fate (Kampinga and Craig, 2010). Molecular chaperones are classified into different families that were originally named for the molecular weight of the founding member. The four main chaperone families in metazoans are Hsp60s, Hsp70s, Hsp90s, and small Hsps (sHsp).

sHsps lack an ATPase domain and therefore generally act as classical holdases (Webster et al., 2019). They can be found in inactive oligomeric complexes that keep them poised to combat an early misfolding event (Santhanagopalan et al., 2018). Stress conditions can activate sHsps to sequester misfolding proteins and protect the substrates from further aggregation and facilitate their re-folding (Biswas and Das, 2004), often in concert with other chaperones, such as Hsp70s (Mogk et al., 2019).

The Hsp70 family consists of heat shock-inducible (e.g., HSP70-1/HSPA1A) and constitutively expressed (e.g., HSC70/HSPA8) members and has highly assorted functions, including the folding of newly synthesized proteins, refolding of misfolded proteins, disaggregation, membrane translocation, endocytosis, and degradation of terminally misfolded proteins. This functional diversity is provided by a myriad of co-chaperones. The Hsp70 core chaperone typically cooperates with a member of the J-domain protein (DNAJ) family and a nucleotide exchange factor (NEF) that regulate the Hsp70 ATPase cycle (Mayer and Bukau, 2005). The DNAJ family expanded from six DNAJs found in *E*. *coli* to 49 in *Homo sapiens* (Finka and Goloubinoff, 2013; Bar-Lavan et al., 2016). This increase in complexity may reflect the evolutionary selection pressure for greater versatility of Hsp70 machines. After being processed by Hsp70s and their co-chaperones, clients can be subsequently handed over to chaperonins and Hsp90 family members.

The ATP-dependent Hsp60 family, also commonly referred to as the chaperonins, is divided into two groups: Group I is generally found in eubacteria, but also in evolutionarily derived mitochondria, and Group II is found in archaea and in the eukaryotic cytosol (Hartl and Hayer-Hartl, 2002). The eukaryotic chaperonin, also known as t-complex 1 (TCP1), or chaperonin containing TCP1 (CCT), is a multiprotein complex composed of two rings with eight different but similar subunits. Driven by ATP-binding and hydrolysis, the subunits open and close a central folding chamber that encapsulates substrate proteins. It is essential as it supports the folding of ~10% of all newly synthesized proteins, in particular actin and tubulin (Yam et al., 2008).

The members of the Hsp90 family are highly conserved and exist in all kingdoms of life except archaea. Similar to the Hsp70 family, there are inducible (e.g., HSP90AA1) and constitutively expressed variants (e.g., HSP90B1) that interact with more than 20 co-chaperones and adaptors thereby regulating a multitude of cellular processes (Taipale et al., 2010; Biebl and Buchner, 2019). Since kinases and steroid hormone receptors are major clients of Hsp90s, they are key regulators of many signaling pathways.

In addition to these main chaperone classes, there are several other types of metazoan chaperones for which a relationship with a particular prion-like protein has been established. Details about these chaperones are given in the respective individual sections.

At first, the role of chaperones in prion-like propagation of misfolded proteins might seem obvious, as the main task of chaperones is to support the correct folding of proteins and protect them from misfolding and aggregation. While this is often the case, there are nonetheless also conflicting results where chaperones have been shown to aggravate protein misfolding or toxicity. Although chaperones may interact with the native state of prion-like proteins also under physiological conditions, we will focus here on the interaction with pathological forms, discussing both the beneficial and detrimental impact chaperones may have on the progression of protein misfolding diseases.

### Prion Diseases

Prion diseases or transmissible spongiform encephalopathies (TSEs) are fatal neurodegenerative diseases that affect humans and animals, including BSE (also known as mad cow disease) in cattle, CWD in deer and elk, scrapie in sheep and goats, and Creutzfeldt-Jakob disease (CJD) in humans (Imran and Mahmood, 2011; Collinge, 2016; Scheckel and Aguzzi, 2018). All prion diseases are characterized by the accumulation of PrP^Sc^ in the central nervous system. Cellular PrP^C^ is a glycosylphosphatidylinositol (GPI)-anchored membrane protein and has the highest expression in neurons of the brain and the spinal cord (Stahl et al., 1987; Harris et al., 1993; Tichopad et al., 2003). Proposed functions of PrP^C^ include the maintenance of synapses and neuroprotective signaling (Westergard et al., 2007).

While the exact function of PrP^C^ remains unclear, it is, however, crucial for the propagation of PrP^Sc^, as mice lacking the *PRNP* gene are resistant to prion infection (Büeler et al., 1993; Sailer et al., 1994; Brandner et al., 1996). Since PrP^C^ is localized on the cell surface, the first interaction and conversion into pathological PrP^Sc^ likely occur at the plasma membrane (Goold et al., 2011). Also, PrP^C^ is endocytosed in a clathrin-dependent manner and delivered from early endosomes and late endosomal multivesicular bodies (MVBs) to lysosomes for degradation. Blocking PrP^C^ endocytosis inhibits the formation of PrP^Sc^, suggesting that conversion also occurs along the endocytic pathway (Borchelt et al., 1992). PrP^Sc^ is rapidly truncated into a C-terminal PrP27–30 protease-resistant core, which is very stable and accumulates in MVBs and lysosomes. Intercellular transmission proceeds *via* exosomes that are derived from intraluminal vesicles (ILVs) of MVBs and are released into the extracellular space through their fusion with the plasma membrane (Fevrier et al., 2004). In tissue culture cells, PrP^Sc^ was also shown to be able to spread within endocytic vesicles through tunneling nanotubes (TNTs), long membranous tubules that connect the cytosol of two cells (Gousset et al., 2009; Zhu et al., 2015).

Hsp70 family genes are upregulated in CJD patients and prion-infected mice (Kenward et al., 1994; Kovács et al., 2001). Furthermore, several models have shown that manipulation of chaperone levels can influence disease progression, which underlines the relevance of chaperones for prion diseases. When mice that lack heat shock factor 1 (HSF1), the primary transcription factor for the expression of numerous chaperones, are exposed to prions, they succumb to the disease about 20% faster than wildtype animals (Steele et al., 2008). Prion disease progression was also accelerated if cytosolic or endoplasmic reticulum (ER) Hsp70s levels were reduced (Park et al., 2017; Mays et al., 2019). In the opposite direction, the data are less clear. While the induction of HSP70 expression slowed the progression of prion phenotypes in *Drosophila* (Zhang et al., 2014), overexpression of HSP70 had no impact on survival times of prion-infected mice (Tamgüney et al., 2008). Thus, further research is necessary to gain insights into the exact mechanisms by which chaperones influence the course of prion diseases to identify effective therapeutic approaches.

### Parkinson’s Disease and Other Synucleinopathies

Accumulation of aggregated α-syn/SNCA is a hallmark of PD and other synucleinopathies (Uversky, 2003, 2011). In PD, Lewy bodies containing aggregated α-syn, occur in a predictable manner, which is classified into six distinct stages based on the location of α-syn inclusions seen in postmortem brains (Braak et al., 2003). These observations have led to the hypothesis, that pathological α-syn may propagate like prions. This idea has gained momentum through observations in PD patients who underwent embryonic neuronal cell transplantation. When examined several years later, these patients showed signs of disease development in the grafted tissue, indicating that pathological α-syn had spread from diseased to healthy tissue (Kordower et al., 2008; Li et al., 2008). Various animal and cell culture models have confirmed the existence of such intercellular dissemination of α-syn (Jucker and Walker, 2013; Vasili et al., 2019).

The presence of numerous different chaperones in Lewy bodies suggests a central role of these proteins in α-syn pathology (McLean et al., 2002). Moreover, the importance of chaperones and in particular J-domain proteins in the disease is also evident through the discovery of respective mutations in genome-wide association studies. For instance, DNAJC6 mutations have been linked to juvenile parkinsonism (Lin and Farrer, 2014), while DNAJC13/RME8 mutations have been associated with familial forms of PD (Vilariño-Güell et al., 2014).

Prevention of α-syn aggregation has been shown with multiple sHsps (HSPB1, HSPB2, HSPB3, HSPB5, HSPB6, and HSPB8), the J-domain proteins DNAJB6 and DNAJB8, and with Hsp70s (Rekas et al., 2004; Outeiro et al., 2006; Bruinsma et al., 2011; Cox et al., 2016; Aprile et al., 2017; Bendifallah et al., 2020; Vicente Miranda et al., 2020). Hsp90s also prevent α-syn aggregation but by specifically interacting with oligomeric species rather than monomers or fibrils (Falsone et al., 2009; Daturpalli et al., 2013). The constitutively expressed HSC70, along with HSPB1 and HSPB5, can also bind α-syn fibrils, and coating of the fibrillar surface reduced toxicity (Waudby et al., 2010; Pemberton et al., 2011; Redeker et al., 2012; Cox et al., 2018).

Chaperones not only interfere with early nucleation and fibril elongation events but are also able to depolymerize mature α-syn fibrils (Duennwald et al., 2012; Gao et al., 2015). This disaggregation function is dependent on the specific cooperation of the core HSC70 with a class B J-domain protein, DNAJB1, and an Hsp110-type NEF, HSPA4/APG-2 (Gao et al., 2015). HSPB5/αB-crystallin can assist in the depolymerization of α-syn fibrils by stimulating the Hsp70 disaggregase (Duennwald et al., 2012). These *in vitro* observations indicated that α-syn disaggregation might be beneficial and cytoprotective since fibrillar α-syn was eventually dissolved. However, a recent study reported the opposite effect *in vivo*. Diminishing disaggregation activity by knocking down the only cytosolic Hsp110-type NEF, HSP-110 significantly reduced the accumulation of toxic α-syn species in *C. elegans* (Tittelmeier et al., 2020). Moreover, α-syn particles generated by the Hsp70 disaggregase were preferred substrates for intercellular transfer. Hence, the Hsp70 disaggregation machinery seems to be involved in the prion-like propagation of α-syn by generating seeding-competent α-syn species, and blocking this activity is beneficial with regard to amyloidogenic substrates (Tittelmeier et al., 2020). While this result seems counterintuitive at first, it is reminiscent of the crucial role of Hsp104 in the propagation of yeast prions, where depletion of Hsp104 leads to a rapid loss of yeast prions (Chernoff et al., 1995). Hsp104 cooperates with the Hsp70 chaperone system and promotes prion replication by extracting monomers from prion fibrils, which leads to their fragmentation and the increased generation of propagons (Jones and Tuite, 2005; Tessarz et al., 2008; Tipton et al., 2008). Hexameric AAA+ Hsp100-type disaggregates such as Hsp104 are absent in metazoans, but the Hsp70 system has evolved to provide this function (Shorter, 2011; Rampelt et al., 2012). While chaperone-mediated disaggregation seems to significantly contribute to the toxicity associated with pathological α-syn, it is essential for the maintenance of cellular proteostasis, as reducing HSP-110 levels compromised the overall cellular protein folding environment (Tittelmeier et al., 2020). For this reason, complete inhibition of this activity is not a suitable intervention strategy. Rather, the modulation of individual isoforms or more specific components may be a promising therapeutic approach. The observed adverse side effects could be minimized, e.g., by the only temporary intake of drugs that inhibit the machinery. Also, the human chaperome is more redundant (there are e.g., three Hsp110-type NEFs compared to only one in *C. elegans*, Brehme et al., 2014) and the reduction of a single-player would probably reduce rather than eliminate disaggregation activity and result in fewer side effects. Nevertheless, more studies are needed to explore the usefulness of this approach.

Another way in which chaperones can help prevent the spreading of α-syn is by facilitating the elimination of aberrant species. Turnover of α-syn can be mediated by the UPS, with the HSC70 co-chaperone carboxyl terminus of Hsp70-interacting protein (CHIP) governing this degradation pathway (Shin et al., 2005). Another process, described as chaperone-mediated autophagy (CMA), also involves HSC70, which targets misfolded α-syn and translocates it into lysosomes for degradation (Cuervo et al., 2004). However, CMA and lysosomal degradation are often impaired in PD (Pan et al., 2008; Chu et al., 2009).

The exact mechanisms of intercellular transfer of α-syn are not yet fully understood, but current research suggests several parallel transmission routes (Brundin and Melki, 2017; Vasili et al., 2019). First, α-syn must exit the donor cell. As a cytosolic protein, α-syn is not released by the conventional secretory pathway *via* the ER and Golgi apparatus. Instead, there is growing evidence that the endo-lysosomal system is involved in α-syn spreading in addition to its role in the degradation of the protein. Endosomal α-syn can be either directly transported to neighboring cells *via* TNTs (Abounit et al., 2016; Rostami et al., 2017; Victoria and Zurzolo, 2017), or it can eventually be released *via* unconventional secretion pathways involving secretory lysosomes or exosomes (Emmanouilidou et al., 2010; Danzer et al., 2012; Ngolab et al., 2017; Tsunemi et al., 2019). Another recently described mechanism for the release of aberrant protein species is the misfolding-associated protein secretion (MAPS) pathway (Lee et al., 2016). Here the ER-associated deubiquitinase USP19 recruits misfolded cytosolic proteins, such as α-syn, to the ER surface and then transfers them to DNAJC5 and HSC70 localized at late endosomes (LEs), which finally fuse with the plasma membrane and release their content to the extracellular space (Fontaine et al., 2016; Xu et al., 2018). Spreading also relies on the uptake of extracellular α-syn into the recipient cell. To this end, clathrin-mediated endocytosis is involved in the uptake of free or exosomal α-syn (Oh et al., 2016; Ngolab et al., 2017). As part of this process, HSC70 cooperates with DNAJC6/Auxillin and an Hsp110-type NEF to disassemble clathrin coats (Sousa and Lafer, 2015). After uptake, misfolded α-syn species must enter the cytosol to be able to template the aggregation of native α-syn in the recipient cell. Indeed, α-syn has been shown to escape from endocytic vesicles by rupturing the endosomal membrane (Flavin et al., 2017).

### Alzheimer’s Disease and Other Tauopathies

#### Tau

More than 20 different neurodegenerative diseases are associated with the progressive accumulation of Tau inclusions in different brain areas and cell types which are collectively referred to as tauopathies, including AD and frontotemporal dementia (FTD; Goedert et al., 2017a). The sequential appearance of Tau aggregates in the brain during disease progression follows a stereotypic distribution pattern, categorized into six “Braak stages” according to the prevalence of Tau pathology in different brain regions (Braak and Braak, 1991; Jucker and Walker, 2013). Intriguingly, the extent of Tau deposition in the different brain regions is a good correlate for the disease stage (Jucker and Walker, 2013). Tau’s capacity to propagate in the brain is further supported by extensive research in mouse models. Injection of recombinant or patient-derived Tau aggregates into mouse brains causes the formation of Tau inclusions both at the injection site and in distant interconnected brain areas (Narasimhan and Lee, 2017). Therefore, it is assumed that seeding-competent Tau material is transported to other parts of the brain in a connectivity-dependent manner where it induces the aggregate formation of native Tau (Goedert et al., 2017a).

Although we are mainly focusing here on the effect of chaperone action on pathological Tau species, it is worth mentioning that under healthy conditions, various chaperones control the homeostasis of native Tau, such as its loading onto microtubules or degradation *via* the proteasome and autophagy pathways (Miyata et al., 2011; Young et al., 2018).

NMR studies with monomeric Tau have identified binding sites for various chaperones that are either close to or within the repeat domains that contribute to the β-sheet structures in amyloid Tau fibrils (Jinwal et al., 2013; Mok et al., 2018). By interacting with this region chaperones can stabilize soluble Tau and thereby prevent its assembly into amyloid fibrils. Several Hsp70 family members, their co-chaperones DNAJA1 and DNAJA2, chaperonin, various sHsps, as well as the extracellular chaperone clusterin (CLU) delay the aggregation of wildtype and aggregation-prone Tau mutants *in vitro* (Patterson et al., 2011; Mok et al., 2018). Additionally, it has been shown that Hsp70s suppress Tau aggregation by stabilizing Tau oligomers to inhibit further seeding (Kundel et al., 2018) and by preventing fibril elongation into larger assemblies (Patterson et al., 2011; Baughman et al., 2018). This prevention of Tau aggregation and fibril growth observed *in vitro* presumably delays disease onset and progression, as supported by studies in *in vivo* models, in which the absence of particular chaperones led to accelerated Tau aggregation and toxicity (Eroglu et al., 2010). In line with this, HSPB1 overexpression decreased Tau levels and rescued the Tau mediated damage in a mouse model (Abisambra et al., 2010). Interestingly, HSP90 stabilizes aggregation-prone conformations of Tau and promotes oligomer formation *in vitro* (Weickert et al., 2020). However, the fate of Tau is highly dependent on the specific HSP90 co-chaperone (Shelton et al., 2017). For instance, overexpression of the co-chaperone FKBP prolyl isomerase 5 (FKBP51) in a mouse model increases Tau oligomers at the cost of fibril formation and at the same time enhances neurotoxicity (Blair et al., 2013).

Chaperones do not only suppress or delay the initial aggregation of monomeric and oligomeric Tau species but are also capable of dissolving Tau fibrils. The aforementioned trimeric human Hsp70 disaggregation machinery (HSC70, DNAJB1, HSPA4) readily disassembles a variety of amyloid Tau aggregates ranging from *in vitro* assembled fibrils to aggregates extracted from a cell culture model to brain material of AD patients (Nachman et al., 2020). Thus, the Hsp70 disaggregation machinery is capable of disaggregating pathologically relevant forms of Tau. Although mainly monomers were released the liberated Tau species were nevertheless seeding-competent and induced self-propagating Tau aggregates in a cell culture model (Nachman et al., 2020). In subsequent research, it needs to be determined whether disaggregation of amyloid Tau fibrils may exacerbate neurotoxicity *in vivo*. However, it is tempting to speculate that chaperone-mediated disaggregation might promote the prion-like propagation of amyloid Tau aggregates and eventually increase the overall amyloid burden *in vivo*, especially considering its effect on α-syn aggregation and toxicity in *C. elegans* discussed above (Tittelmeier et al., 2020). Interestingly, the co-chaperone DNAJB4 can substitute for DNAJB1 in the Hsp70 disaggregase, while class A J-domain proteins are unable to support disaggregation of Tau, indicating specificity, but also a certain redundancy in the recognition of amyloid Tau (Nachman et al., 2020). Interfering with the specific interaction between these class B J-domain proteins and amyloid fibrils could be an effective treatment strategy to reduce unfavorable amyloid disaggregation without affecting the processing of other substrates.

As Tau is a cytoplasmatic protein that deposits intracellularly, the spreading of Tau requires release and uptake of seeding-competent Tau material from the cytosol of donor and receiving cells. Similar to α-syn, Tau is also a substrate of the MAPS pathway (Fontaine et al., 2016; Lee et al., 2016; Xu et al., 2018), where HSC70 together with its co-chaperone DNAJC5 promotes the release of Tau into the extracellular space both in cell culture and in a mouse model. However, it remains unknown which Tau species get released *via* this pathway and whether this material can then seed the aggregation of native Tau molecules in recipient cells. Following its release, extracellular Tau can then be taken up by neighboring cells by similar routes described for α-syn (Goedert et al., 2017b). However, it is not yet clear to what extent clathrin-mediated endocytosis and the chaperones involved could contribute to Tau propagation (Yoshiyama et al., 2007; Holmes et al., 2013; Calafate et al., 2016).

#### Aβ

AD is characterized by the deposition of both intracellular Tau aggregates and extracellular senile plaques consisting of the Aβ peptide in the brain (Goedert and Spillantini, 2006). The Aβ peptide is generated by endoproteolytic cleavages within the transmembrane protein amyloid-β precursor protein APP (De Strooper and Annaert, 2010). Similar to the stereotypical deposition of Tau and α-syn aggregates, the appearance of Aβ plaques follows a predictable pattern that sequentially affects certain areas of the brain during disease progression (Jucker and Walker, 2013). The prion-like behavior of Aβ has been confirmed in numerous rodent models (Meyer-Luehmann et al., 2006; Eisele et al., 2010). Moreover, it has been shown that cadaveric pituitary-derived human growth hormone, which was contaminated with Aβ seeds, caused a plaque-like pathology in treated patients, suggesting an iatrogenic transmission of Aβ pathology (Jaunmuktane et al., 2015; Purro et al., 2018).

*In vitro* studies have identified several cytosolic chaperones, such as the sHsps HSPB1, HSPB5, HSPB6, and HSPB8, the J-domain protein DNAJB6, as well as chaperonin that suppress Aβ aggregation, either by inhibiting initial aggregation or recruiting oligomeric species into larger structures (Lee et al., 2006; Wilhelmus et al., 2006; Shammas et al., 2011; Månsson et al., 2014a; Mangione et al., 2016; Vilasi et al., 2019). As the sequestration of oligomers reduces the number of particles that can act as seeds this mechanism could help to limit the incorporation of monomers by templated misfolding. Almost all amino acids in the Aβ peptide are incorporated into its amyloid fold (Kollmer et al., 2019). In contrast, larger proteins, such as α-syn and Tau have N- and C-terminal extensions forming a fuzzy coat around their amyloid cores (Scheres et al., 2020; Schweighauser et al., 2020). The lack of available binding sites flanking the amyloid core is probably the reason why the canonical Hsp70 chaperone machinery does not interact with Aβ assemblies (Kakkar et al., 2014; Wentink et al., 2019). In contrast, the mitochondrial chaperonin HSPD1 can bind to Aβ oligomers, which reduces Aβ-mediated neurotoxicity by preventing Aβ oligomers from interacting with membranes (Marino et al., 2019; Vilasi et al., 2019). It is tempting to speculate that this function might be conserved by the homologous cytosolic chaperonin CCT, which could help to reduce Aβ toxicity by preventing disruption of intracellular membranes (Julien et al., 2018). Yet, the physiological relevance of these findings regarding cytosolic chaperones remains to be investigated. Aβ aggregates form in the endosomal-lysosomal pathway and plaques deposit in the extracellular space (Peric and Annaert, 2015). However, a contribution of cytoplasmic Aβ oligomers to amyloid toxicity and transmission has been demonstrated in cell culture models (Nath et al., 2012). It will be interesting to test whether cytosolic chaperones directly interact with these cytoplasmic Aβ species and modulate their properties *in vivo*.

The amyloid formation can also be accelerated by secondary nucleation events on amyloid surfaces as their interaction with monomers catalyzes the formation of new seeds (Törnquist et al., 2018). By shielding such surfaces, the BRICHOS domain inhibits Aβ aggregation *in vitro* by interfering with oligomerization and secondary nucleation (Willander et al., 2012). The BRICHOS domain is found in the proteins Bri2 and Bri3 that co-localize with extracellular Aβ plaques in the early stages of disease (Del Campo and Teunissen, 2014; Dolfe et al., 2018), which hints to a potential role of these proteins in containing the spreading of pathology by shielding the plaques. The extracellular chaperone CLU is a well-established genetic risk factor for AD (Foster et al., 2019). CLU prevents Aβ aggregation *in vitro* by sequestering and stabilizing oligomeric species (Narayan et al., 2012; Beeg et al., 2016). While several studies have found CLU reduces the uptake of Aβ oligomers and fibrils into neurons and microglia in cell culture models (Nielsen et al., 2010; Mulder et al., 2014), others have observed enhanced Aβ uptake in the presence of CLU (Yeh et al., 2016). A contribution of CLU to cellular uptake of Aβ would directly impact Aβ transmission. Thus, to be able to exploit CLU as a potential therapeutic target, it is essential to further explore its role in the intercellular dissemination of Aβ.

### Polyglutamine Diseases

There are nine different adult-onset autosomal dominantly inherited diseases that are caused by an expansion of a trinucleotide (CAG) repeat encoding a polyglutamine (polyQ) tract, including HD and six forms of spinocerebellar ataxia (Lieberman et al., 2019). The disease-associated proteins are not related to each other, but they all contain a polyQ tract with a length of 10–35 glutamine repeats in healthy individuals. A stretch of 40 or more glutamines will eventually cause disease, with a longer expansion correlating with an earlier age of onset (Scherzinger et al., 1999). PolyQ expansion is associated with the formation of amyloid aggregates, which can be localized in the nucleus or in the cytoplasm (Scherzinger et al., 1997; Reddy et al., 1999). HD is the most prevalent of these diseases, where polyQ expansion occurs in HTT.

Genetic screens for modifiers of polyQ aggregation have identified several chaperones (Krobitsch and Lindquist, 2000; Nollen et al., 2004; Silva et al., 2011). Furthermore, activation of HSF1 ameliorates the toxicity of polyQ *in vivo* (Fujikake et al., 2008; Kumsta and Hansen, 2017). Many sHsps, including HSPB1, HSPB4, HSPB6, HSPB7, HSPB8, and J-domain proteins, including DNAJB1, DNAJB2, DNAJB6, and DNAJB8, were shown to prevent the initial aggregation of polyQ (Kazemi-Esfarjani and Benzer, 2000; Willingham et al., 2003; Carra et al., 2005; Hageman et al., 2010; Vos et al., 2010; Labbadia et al., 2012; Månsson et al., 2014b), while the chaperonin CTT prevented polyQ aggregation by capturing smaller oligomeric species (Tam et al., 2009; Shahmoradian et al., 2013). DNAJB6 emerges as a key protective co-chaperone for polyQ containing sequences and has been shown to very efficiently inhibit the primary nucleation step in polyQ amyloid formation by directly binding to the polyQ tract (Gillis et al., 2013; Kakkar et al., 2016). Moreover, recent research revealed that during differentiation of pluripotent stem cell lines from HD patients into neurons, there is a loss of expression in DNAJB6, which leads to aggregation of polyQ (Thiruvalluvan et al., 2020). This could explain why pathological aggregates are predominantly present in neurons and why stem cells are protected.

The Hsp70 system plays a multifaceted role in polyQ aggregation and toxicity. HSP70 is involved in the prevention of polyQ aggregation alone (Muchowski et al., 2000; Monsellier et al., 2015), as well as in collaboration with the J-domain protein DNAJB1 (Wacker et al., 2004). HSP70 is also capable of sequestering smaller polyQ species into larger non-toxic fibrillar structures thereby preventing their toxic interaction with other cellular components (Behrends et al., 2006). This function is mediated by HSP70 with the help of CCT and a J-domain protein (Behrends et al., 2006). Short Q-rich peptides can also shield the interactive surfaces of polyQ proteins, altering the interaction of other prion-like proteins, directing them into nontoxic aggregates (Ripaud et al., 2014).

The HSC70-DNAJB1-HSPA4 disaggregation machinery is not only able to disintegrate α-syn and Tau fibrils as described above (Gao et al., 2015; Nachman et al., 2020), but also disentangle polyQ fibrils (Duennwald et al., 2012; Scior et al., 2018), which demonstrates the high versatility of this chaperone machinery. Similar as in the case of α-syn, compromising this disaggregase leads to fewer aggregates and rescues the toxicity of polyQ in *C. elegans* (Tittelmeier et al., 2020). This is in agreement with observations in yeast, where deletion of the yeast disaggregase Hsp104 also leads to a decrease in polyQ aggregates (Krobitsch and Lindquist, 2000). These data imply that chaperone-mediated disaggregation can handle many different types of amyloid aggregates and as a result, it could play a central role in the prion-like propagation of aggregates in many diseases, rendering it a highly attractive therapeutic target.

While polyQ aggregates can replicate by inducing the aggregation of native proteins through a templated seeding mechanism, like the other prion-like proteins, the relevance of intercellular spreading of protein aggregates in disease pathogenesis is unclear, especially because of the strong genetic component of these diseases. There is currently also no evidence for the involvement of chaperones in the intercellular spreading of polyQ proteins besides a potential indirect effect on vesicle trafficking.

However, multiple studies are implying non-cell-autonomous effects in these diseases, such as excitotoxicity, where neurons die as a result of disturbances in the surrounding supporting cells. Models, where polyQ is expressed exclusively in the most vulnerable neurons, fail to elicit many disease symptoms, which are seen when polyQ is expressed not only in neurons but also in glial cells (Gu et al., 2005; Sambataro and Pennuto, 2012). This suggests that some aspects of disease pathology are due to non-cell-autonomous toxicity. For example, the selective vulnerability of neurons has been linked to aberrant activation of glutamate. Normally, this is regulated by the uptake of glutamate into glial cells, however, this process is altered in glial cells expressing disease-associated polyQ proteins (Liévens et al., 2001). Targeting this interconnection seen between neurons and glial cells may be a practical goal in the treatment of these diseases. Recently, this idea was explored using a *Drosophila* model with polyQ expressed in neurons and DNAJB6 expressed in glial cells. The exclusive expression of DNAJB6 in glial cells results in the non-cell-autonomous protection against neurodegeneration and prolongs lifespan (Bason et al., 2019). A deeper understanding of how chaperones could alleviate the non-cell-autonomous effects of prion-like proteins could reveal an exciting new therapeutic approach.

### Amyotrophic Lateral Sclerosis

ALS is a fatal, rapidly progressing disease characterized by the degeneration of upper and lower motor neurons. Typically, motor symptoms manifest at mid-adulthood and begin in a restricted part of the body, which varies from patient to patient and then spreads to neighboring areas. This implies a prion-like pathomechanism based on neuronal connectivity (Ravits and La Spada, 2009; Sibilla and Bertolotti, 2017). The speed at which symptoms spread from one area to another correlates with disease duration (Ravits, 2014).

In motor neurons of patients with both familial and sporadic forms of the disease, protein inclusions have been found postmortem that usually contains either SOD1 or ubiquitinated TDP-43 (Kwong et al., 2007). Moreover, various missense mutations have been identified in SOD1, TDP-43, and FUS, as well as a hexanucleotide repeat expansion in C9orf72, which increase the aggregation propensity of these proteins and are associated with familial ALS (fALS) accounting for 10% of all ALS cases (Sibilla and Bertolotti, 2017). Also, exome sequencing of a large ALS patient cohort identified several mutations in the J-domain protein DNAJC7 that led to reduced protein levels in patient-derived fibroblasts (Farhan et al., 2019). This finding directly links chaperone activity to ALS etiology. Although further work is required to elucidate how the loss of DNAJC7 function causes ALS, this underlines the importance of the PQC system for these protein misfolding diseases.

#### SOD1

The prion-like behavior of SOD1 has been established over the last decade employing *in vitro* and cell culture systems as well as mouse models. Recombinant SOD1 aggregates act as seeds accelerating the aggregation of natively folded SOD1 *in vitro* (Chattopadhyay et al., 2008). In cell culture, SOD1 aggregates can spread intercellularly within exosomes or *via* direct release into the extracellular space (Münch et al., 2011; Grad et al., 2014; Silverman et al., 2016). The released SOD1 species are taken up from the medium by the receiving cells *via* micropinocytosis (Münch et al., 2011). Subsequently, the SOD1 aggregates escape into the cytoplasm where they seed aggregation of the native SOD1 molecules forming self-propagating foci (Münch et al., 2011). Also, SOD1 aggregate pathology can be transmitted between mice through the injection of brain homogenate (Ayers et al., 2014).

Members of the Hsp70 family and their J-domain partner protein and sHsp chaperones have been found to colocalize with SOD1 inclusions in patient tissues and rodent ALS models and to interact with mutant SOD1 in cell culture models, indicating that aggregated SOD1 is recognized as a substrate by the PQC system (Shinder et al., 2001; Watanabe et al., 2001; Howland et al., 2002; Liu et al., 2005; Matsumoto et al., 2005). However, even though the sHsps HSPB1 and HSPB5 reduce SOD1 aggregation *in vitro* (Yerbury et al., 2013), overexpression of HSPB1 in a SOD1^G93A^ mouse model only delayed the onset of motor symptoms and did not affect the overall survival of these mice (Sharp et al., 2008), suggesting that increasing the levels of individual chaperones does not always lead to a beneficial outcome. Accordingly, while increased levels of Hsp70 family members reduced SOD1 aggregation and toxicity in cultured mouse primary motor neurons expressing SOD1^G93A^ (Bruening et al., 1999), this had no impact on either survival or onset of motor symptoms in several SOD1 mutant mouse models, including SOD1^G93A^ (Liu et al., 2005). Interestingly, Serlidaki et al. (2020) have recently demonstrated in cell culture that the effect of Hsp70s on the aggregation of the SOD1^A4V^ mutant depends on the particular Hsp70 variant, which is overexpressed. While HSPA1A suppresses SOD1 aggregation, its close homolog HSPA1L enhances aggregate formation. The differences in client fate seem to result from the fact that Hsp110-type co-chaperones prefer to interact with HSPA1A rather than HSPA1L (Serlidaki et al., 2020). More specifically, HSPA1A requires the NEF HSPA4 (APG-2) to inhibit SOD1 aggregation, while the aggregation promoting activity of HSPA1L does not depend on this NEF. However, it remains unknown which specific molecular mechanisms determine the affinity of Hsp110 family members for different Hsp70s in the cellular context.

It is therefore not surprising that NEF overexpression has also varying consequences. Overexpression of BAG1 in SOD1^G93A^ transgenic mice did not improve their survival or the onset of ALS-like phenotypes (Rohde et al., 2008). In contrast, overexpression of the Hsp110-type NEF HSPA4L (APG-1) in SOD1^G85R^ transgenic mice extended the lifespan of these animals (Nagy et al., 2016). However, the molecular mechanism underlying the beneficial effect of HSPA4L overexpression remains unclear. While the authors speculate that elevated levels of HSPA4L might lead to an increase in Hsp70 disaggregation capacity, the opposite is also conceivable. It has been shown that excessive concentrations of NEFs can poison the Hsp70 system (Nollen et al., 2000; Kampinga and Craig, 2010; Gao et al., 2015). Usually, NEFs function in sub-stoichiometric amounts relative to Hsp70, and increasing their concentrations beyond this optimal ratio will overstimulate the ATPase cycle and consequently lead to too rapid dissociation of the substrate. As a result, the client protein would be released prematurely. Consequently, NEF overexpression would disrupt rather than promote the Hsp70 function. Further research is, therefore, necessary to shed light on this important aspect.

Currently, there is no evidence that chaperones are directly involved in the intercellular transmission of SOD1. However, chaperones are capable of modulating SOD1 aggregation and toxicity, which could at least indirectly affect the spreading. Moreover, since it has been shown that the sHsp HSPB8 together with HSC70, BAG3, and CHIP, mediates the autophagic degradation of misfolded SOD1 and thus directs it into the endo-lysosomal pathway (Crippa et al., 2010), this could promote the dissemination of SOD1 assuming that it follows similar routes as α-syn and Tau (Uemura et al., 2020).

Future research is needed to gain a more comprehensive picture of which sets of chaperones and co-chaperones act together to suppress or enhance SOD1 aggregation. Furthermore, it is necessary to evaluate the effect of modifying individual chaperone levels in *in vivo* models to predict the overall effect on SOD1 pathology.

#### TDP-43

TDP-43 is the main component of the characteristic protein inclusions in the central nervous system (CNS) of patients suffering from sporadic ALS (sALS) (Neumann et al., 2006). TDP43 pathology can be observed in about 95% of all sALS cases but is also frequently found in other neurodegenerative diseases, such as frontotemporal lobar degeneration (FTLD), in which ubiquitin-positive Tau-negative TDP-43 inclusions occur. These diseases are collectively referred to as TDP-43 proteinopathies (Arai et al., 2006).

Although the disease-associated aggregation and spreading of TDP-43 have not yet been studied in as much detail as other prion-like proteins, such as Tau or α-syn, there is nevertheless strong indication for a prion-like pathomechanism of TDP-43. The progressive spreading of TDP-43 pathology between interconnected brain areas upon injection of patient-derived pathological TDP-43 was demonstrated in a mouse model (Porta et al., 2018). In cell culture models, both recombinant TDP-43 aggregates and patient-derived detergent-insoluble TPD-43 are taken up from the medium and seed the aggregation of endogenous TDP-43 in the cytoplasm (Furukawa et al., 2011; Nonaka et al., 2013). The intercellular transmission of seeding-competent TDP-43 species in these model systems occurs at least in part *via* exosomes (Nonaka et al., 2013; Iguchi et al., 2016). Intriguingly, exosomes containing seeding-competent TDP-43 are also present in the cerebrospinal fluid (CSF) of ALS patients, which could contribute to the spreading of pathology during disease progression (Iguchi et al., 2016).

TDP-43 is a client of several chaperone families. Consequently, enhanced chaperone expression due to HSF1 activation reduces TDP-43 aggregation and restores TDP-43 solubility (Chen et al., 2016; Wang et al., 2017). While Chen et al. (2016) attributed this observation to the induction of Hsp70s and the co-chaperone DNAJB2a, a similar approach by Wang et al. (2017) identified DNAJB1 and HSPB1 as the major downstream factors of HSF1. *In vitro* assays will be required to identify the specific molecular mechanisms by which each of these chaperones interact with TDP-43 and at what stage during TDP-43 aggregation they act.

Also, TDP-43 is degraded by chaperone-assisted selective autophagy (CASA), where the sHsp HSPB8 works together with HSC70, BAG3, and CHIP to deliver substrates to autophagy. Inducing HSPB8 in a cell culture model increases TDP-43 turnover and overexpression of the *Drosophila* HSPB8 ortholog suppresses TDP-43-mediated neurotoxicity (Crippa et al., 2010, 2016; Gregory et al., 2012). The presence of TDP-43 in the lysosomal fraction isolated from the rodent brain also indicates autophagy-mediated turnover (Ormeño et al., 2020). Moreover, both recombinant wildtype TDP-43 and an aggregation-prone mutant are degraded by isolated lysosomes *in vitro* and TDP-43 is a substrate of CMA interacting with the major CMA components in cell culture (Huang et al., 2014; Ormeño et al., 2020). Although TDP-43 aggregation upregulates CMA, it simultaneously disturbs the membrane integrity of LAMP2A-positive lysosomes compromising the autophagolysosomal pathway (Ormeño et al., 2020). It is therefore highly likely that the disruption of lysosomal membranes leads to the release of seeding-competent TDP-43 species and thus contributes to TDP-43 spreading similarly as previously shown for α-syn and Tau (Flavin et al., 2017). In addition to α-syn and Tau, TDP-43 is also a client of the MAPS pathway (Fontaine et al., 2016), but whether this HSC70/DNAJC5-dependent release of TDP-43 contributes to the spreading of TDP-43 pathology in ALS and FTD patients remains to be shown.

Taken together, TDP-43 shares significant characteristics of a prion-like protein, and chaperones are involved at several key steps of TDP-43 turnover, which could be critical for the propagation of TDP43 pathology. However, to obtain a comprehensive picture of the specific mechanisms by which individual chaperones or combinations of chaperones influence TDP-43 aggregation, *in vitro* studies investigating the direct effect of chaperones on TDP-43 aggregation kinetics using recombinant proteins are required. This would be of great value for the discovery of potential drug targets.

#### FUS and C9orf72

While for the other ALS-related proteins such as SOD1 and TDP-43 a prion-like behavior is well established, data indicating an intercellular spreading mechanism for FUS and C9orf72-derived DPRs is only recently emerging (Nomura et al., 2014; Feuillette et al., 2017; Zhou et al., 2017; Morón-Oset et al., 2019). However, since chaperones are known to be able to modulate the toxicity of FUS and C9orf72 (Deng et al., 2015; Cristofani et al., 2018), it is highly likely that they would also affect their transmission in one way or the other.

#### The Role of Chaperones in the Homeostasis of Membraneless Compartments

Over the last decade, it has become increasingly clear that there is a relationship between neurodegenerative diseases, in particular ALS and FTD, and abnormal formation of membraneless cellular compartments (Alberti and Dormann, 2019). Prion-like proteins, including TDP-43 and FUS, contain intrinsically disordered domains that can undergo liquid-liquid phase separation (LLPS), which is crucial for the formation of membraneless cellular compartments, such as nucleoli and stress granules (SGs; Alberti and Dormann, 2019). During phase separation a homogenous solution of macromolecules partitions into two distinct phases; specific macromolecules accumulate to form a denser phase, while the remaining phase is depleted of these components (Hyman et al., 2014). This dense phase is not solid but behaves liquid-like, i.e., it can undergo droplet fusion and is characterized by high internal dynamics as the enriched components rapidly move in and out of this phase (Alberti et al., 2019).

Under healthy conditions, TDP-43 and FUS co-localize with RNA-containing SGs, which are normally formed transiently upon cellular stress and rapidly dissolve as the stress subsides (Aulas and Velde, 2015). However, disease-associated mutations in these proteins alter their phase separation characteristics towards more solid states, which has been shown to impede SG dissociation and promote the maturation from liquid droplets into immobile aggregates both *in vitro* and in cell culture models (Patel et al., 2015). Moreover, non-RNA-binding proteins implicated in other proteinopathies, such as Tau (Wegmann et al., 2018) and α-syn (Ray et al., 2020) may also undergo LLPS, suggesting that this could represent a pathway for the formation of amyloid aggregates in general.

**Figure 3:**
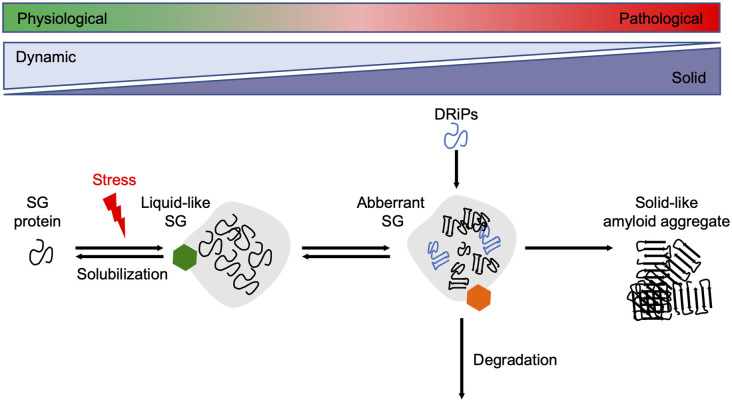
Chaperones maintain SG dynamics. Proteins containing intrinsically disordered domains can undergo phase separation upon cellular stress, such as heat stress, and form liquid-like SGs. Under healthy conditions, the SGs are dissolved when the stress subsides. Chaperones (green hexamer) such as Hsp70s, sHsps, and DNAJB6 are involved in the resolubilization process and thus regulate SG homeostasis. Disease-associated mutations in SG associated proteins, the accumulation of DRiPs, or prolonged exposure to stress conditions reduce the fluidity of SGs, leading to a more solid-like structure that promotes amyloid formation over time. The HSPA1A-BAG3-HSPB8 chaperone network (orange hexamer) targets aberrant SGs for degradation. SG, stress granule.

Since reduced dynamics and a more rigid consistency within phase-separated compartments ultimately promote amyloid fibril assembly, their formation and disintegration need to be tightly controlled by the cellular PQC machinery (Alberti and Dormann, 2019). Several *in vitro* and cell culture studies have shown that molecular chaperones are recruited into SGs and regulate SG formation and stability (Figure 3).

Under normal conditions, DNAJB6 and HSPA1A bind to TDP-43 and control its accumulation into SGs (Udan-Johns et al., 2014). However, upon heat shock, the availability of these chaperones becomes limited, which favors the formation of insoluble TDP-43 aggregates. DNAJB6 also helps to resolve nuclear TDP-43 SGs which form under healthy conditions in response to a heat shock (Stein et al., 2014). In this study, a DNAJB6 harboring a mutation in the G/F domain is unable to promote SG dissolution, leading to the persistence of TDP-43 aggregates. The fact that a mutation in DNAJB6 is associated with limb-girdle muscular dystrophy which in turn is accompanied by pathological TDP-43 aggregates, indicates a protective function of DNAJB6 against dysregulated TDP-43 SG formatio (Harms et al., 2012).

In addition to TDP-43, aggregation-prone SOD1 variants can also cause SGs to transition to a more solid state (Mateju et al., 2017). In this study, Hsp70 family members and the sHsp HSPB1 were enriched in SOD1 containing SGs. Likewise, HSPB1 regulates the LLPS behavior of FUS depending on the cellular stress state, thus preventing aberrant FUS amyloid formation within SGs (Liu et al., 2020). Moreover, defective ribosomal products (DRiPs), i.e., prematurely terminated nascent polypeptides, accumulate in cytosolic SGs leading to a reduction in SG fluidity (Seguin et al., 2014; Ganassi et al., 2016). A chaperone complex consisting of HSPA1A, BAG3, and HSPB8 monitors SG composition and mediates the degradation of DRiPs to restore SG dynamics (Ganassi et al., 2016).

The involvement of chaperones in regulating the dynamics of membraneless compartments is further supported by their function in the nucleolus. The phase-separated nucleolus serves as a PQC compartment which sequesters misfolded protein species (Mediani et al., 2019). Prolonged exposure to stress or a failure to dissolve the liquid-like phase causes proteins in the nucleolus to transition into an amyloid state (Azkanaz et al., 2019; Frottin et al., 2019; Mediani et al., 2019). Their resolubilization during the recovery period depends on the refolding activity of Hsp70 family members that re-localize to the nucleolus (Audas et al., 2016; Azkanaz et al., 2019; Frottin et al., 2019; Mediani et al., 2019).

In addition to conventional chaperones, several other proteins exhibit chaperone-like activity as they suppress abnormal phase separation of certain RNA-binding proteins. For example, the nuclear import receptor transportin-1 (TNPO1) shifts disease-associated FUS variants to a more dispersed state thus preventing their assembly into granules that inhibit local mRNA translation within the axonal compartment (Qamar et al., 2018).

Taken together, chaperones control properties of phase-separated compartments by modulating their internal dynamics during physiological and stress conditions. They are vital to maintaining their liquid-like state by mediating their disassembly or autophagic degradation (Alberti et al., 2017). This surveillance mechanism prevents abnormal aggregation and amyloid formation within membraneless organelles and thus serves as a cellular defense strategy in protein folding diseases to delay disease onset.

## Conclusions and Future Perspectives

**Figure 4:**
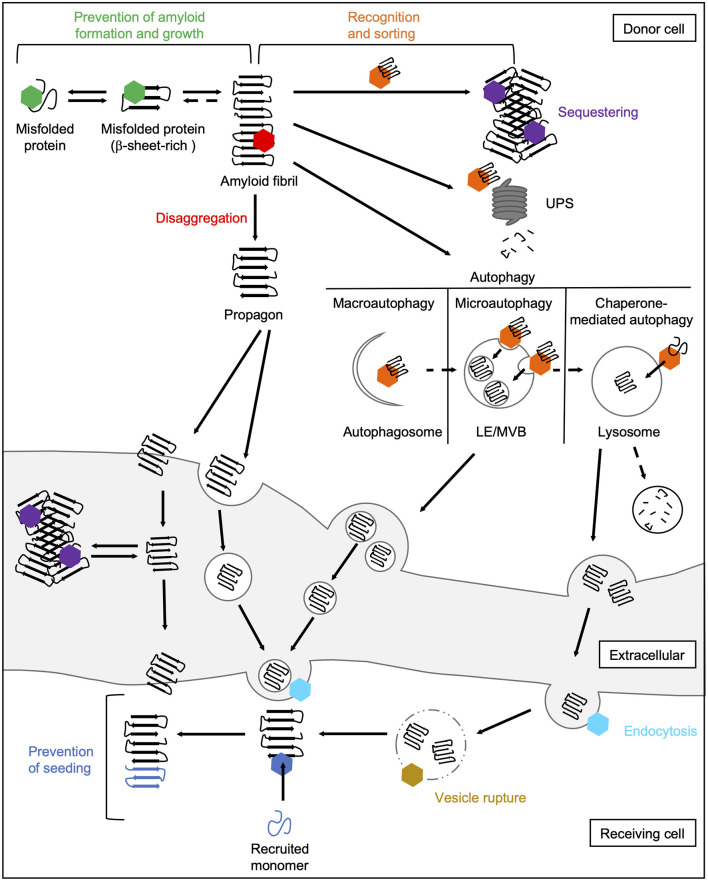
Chaperone interactions during prion-like propagation of disease proteins. The folding and refolding activity of chaperones helps destabilized or misfolded protein species to resume their native state. These transient interactions with misfolded proteins or small oligomers prevent the formation of a seeding-competent propagon (green hexamer). In contrast, the Hsp70 disaggregation machinery can fragment large fibrils leading to the formation of smaller seeding- and spreading-competent species (red hexamer). Chaperones also recognize and sort terminally misfolded forms (orange hexamer) and either mediate their sequestration into an inert deposit (purple hexamer) or deliver them to degradation pathways. Sequestration may reduce the accessibility of fibril ends and thus prevent the further incorporation of native proteins into the amyloid fibril. Extracellular chaperones can also sequester amyloidogenic proteins into large deposits making uptake into receiving cells more difficult. The delivery of amyloidogenic proteins to macroautophagic isolation membranes for their selective clearance is mediated by HSC70 and an sHsp, HSPB8, together with the NEF BAG3 (Gamerdinger et al., 2009). In microautophagy, constitutively expressed HSC70 targets substrates to LEs/MVBs (Sahu et al., 2011). In CMA, HSC70 translocates clients directly across the lysosomal membrane (Tekirdag and Maria Cuervo, 2018). Lysosomes and MVBs can fuse with the plasma membrane releasing either free proteins or exosomes to the extracellular space. In the receiving cell, HSC70 and DNAJC6 are involved in the internalization of misfolded proteins *via* clathrin-mediated endocytosis by uncoating clathrin-coated vesicles (light blue hexamer; Sousa and Lafer, 2015). By rupturing the endosomal membrane, disease-associated proteins are released from the endocytic vesicle into the cytosol (Flavin et al., 2017), which might be prevented by lysosomal or cytosolic Hsps (yellow hexamer). In the cytosol of the receiving cell, chaperones can finally interfere with the seeding of naïve species by the released propagon (dark blue hexamer). CMA, chaperone-mediated autophagy; LE, late endosome; MVB, multivesicular body; NEF, nucleotide exchange factor; UPS, ubiquitin-proteasome system.

The main task of molecular chaperones is to maintain cellular proteostasis. Interactions of chaperones with abnormal protein species are therefore aimed to remove them. In principle, the following basic mechanisms of action of chaperones during prion-like propagation of disease proteins can be distinguished (Figure 4). On the one hand, chaperones can stabilize misfolded protein species, thus preventing their further accumulation (Hartl et al., 2011). Also, they can dissociate protein aggregates by extracting individual monomers (Mogk et al., 2018). Both these processes can eventually help the substrates to regain their native folding state. If this does not succeed, chaperones can control the sequestration of misfolded protein species in a way that prevents harmful interactions with the rest of the proteome (Miller et al., 2015). Finally, they can also mediate their degradation by the UPS (Kästle and Grune, 2012) or autophagy (Menzies et al., 2017). Besides their role in eliminating abnormal protein species, chaperones are also involved in multiple cellular processes, such as endocytosis (Sousa and Lafer, 2015).

Although all chaperone activities are generally “well-intentioned” and many of them have purely positive effects, some also have disadvantages. For instance, the action of the Hsp70 disaggregation machinery can have two outcomes, one beneficial and one harmful. This chaperone system is supposed to dissolve protein aggregates and in the case of amorphous aggregates that are not seeding-competent, it usually accomplishes their dissociation (Rampelt et al., 2012; Tittelmeier et al., 2020). In the case of amyloid substrates, however, this activity seems to liberate more toxic and seeding- and spreading-competent species (Nachman et al., 2020; Tittelmeier et al., 2020), which accelerates prion-like propagation. Also, delivery to the UPS or autophagy is intended to degrade misfolded proteins, but if this fails, these pathways could also generate seeding- and spreading-competent fragments of disease proteins or, in the case of autophagy, facilitate their delivery to neighboring cells. The respective outcome might depend on the state of the cellular proteostasis network. While in young individuals the proteostasis capacity is high and e.g., the products of the disaggregation reaction can either be refolded or degraded, in older individuals the proteostasis capacity is increasingly impaired and the disaggregated material can no longer be efficiently removed. Moreover, chaperones are also involved in trafficking pathways, which are linked to the intercellular spreading of prion-like proteins. Thus, chaperones can also contribute indirectly to the dissemination of propagons by sustaining the cellular pathways required for cell-to-cell spreading, which are hijacked by disease proteins.

A cautionary note is advised when interpreting results from investigating the impact of chaperones. Due to the interconnectivity of the proteostasis network, manipulating expression levels of one chaperone can have unforeseen effects. On the one hand, inhibition of a central chaperone can lead to compensatory upregulation of other chaperones within the network (Sannino et al., 2018). On the other hand, high concentrations of certain chaperones can also have an inhibitory effect. For example, both *in vitro* and *in vivo* work suggest that not only low levels of the HSP110 NEF, but also too high levels can poison the HSP70 disaggregation activity (Nollen et al., 2000; Kampinga and Craig, 2010; Rampelt et al., 2012). Therefore, it would be very beneficial for future studies to characterize not only the expression level of the protein of interest but also the fluctuations of the entire chaperone network; this would allow a more differentiated interpretation of the results.

Taken together, the studies discussed here show that chaperones play an ambivalent role in neurodegenerative diseases. When considering chaperone therapy, it is therefore important to bear in mind that chaperone action is not *per se* beneficial in the context of proteinopathies. Nevertheless, the recent literature established a strong relationship between molecular chaperones and the propagation and spreading of prion-like proteins, suggesting that chaperones are a promising therapeutic target to interfere with the progression of neurodegenerative diseases. While boosting chaperone activity to prevent the initial aggregate formation in early phases of the disease would be the most effective strategy, this might not be feasible as these initial aggregation events usually remain undetected for a long period. During more advanced disease stages, when the proteostasis capacity is already significantly impaired, it might therefore be more effective to interfere with specific chaperone activities to prevent the dissemination or generation of propagons. Since it is not yet fully understood which aggregate species (oligomers, prefibrillar assemblies, or amyloid fibrils) mediate neurotoxicity and which specific variants spread from cell to cell during disease progression (and whether these are the same or distinct species; Ries and Nussbaum-Krammer, 2016), more research is needed to determine which specific chaperone actions are overall beneficial or detrimental *in vivo* as this will determine therapeutic strategies.

Another important aspect to consider in chaperone therapy is that the fate of a certain amyloidogenic protein species depends not only on a single chaperone but also on its interactions with various co-chaperones. Furthermore, it is also determined by the cellular environment. These findings emphasize the complexity within the chaperone network *in vivo*, which cannot be inferred easily from *in vitro* data. More research using *in vivo* models is therefore required to fully understand how chaperone cooperation ultimately determines the fate of certain aggregate species.

Finally, proteinopathies are characterized by disease-specific patterns of neurodegeneration, which mainly affect certain cell types and brain regions during the disease, while others are spared (Jackson, 2014; Fu et al., 2018). There is growing evidence that not only prions but also other prion-like proteins can form fibrils with different conformations, so-called strains, which can affect different brain regions to different degrees (Jackson, 2014; Melki, 2015). These observations raise the question of whether differences in the proteostasis capacity of these cells could be the reason for this selective susceptibility (Jackson, 2014; Labbadia and Morimoto, 2015). In yeast, chaperone activity helps to maintain the conformational diversity of prion strains (Stein and True, 2014; Killian et al., 2019). Thus, it is tempting to speculate that mammalian chaperones could as well promote the structural identity of amyloid conformers and contribute to the amplification of disease-specific strains by generating distinct seeding-competent propagons. Whether and which role differences in the chaperone repertoire play in this process is still completely unclear and an exciting question for the future.

## Author Contributions

JT, EN, and CN-K: conceptualization, investigation (literature review), writing—original draft preparation, and writing—review and editing. JT: visualization. CN-K: supervision, project administration, and funding acquisition. All authors contributed to the article and approved the submitted version.

## Conflict of Interest

The authors declare that the research was conducted in the absence of any commercial or financial relationships that could be construed as a potential conflict of interest.
